# General Medicine as the Clinical Bridge: A Systematic Literature Review of Whole-Person Care, Multimorbidity Management, and Health System Integration

**DOI:** 10.7759/cureus.111077

**Published:** 2026-06-18

**Authors:** Suguna L, Amit Nampalliwar, Nilofer Bano Isa Patel, Megha Garg, Mohanakrishnan B, Hairya Ajaykumar Lakhani

**Affiliations:** 1 Department of General Medicine, Sri Siddhartha Institute of Medical Sciences and Research Center, Sri Siddhartha Academy of Higher Education, Bangalore, IND; 2 Department of Pathology, Government Ayurveda College and Hospital, Bilaspur, IND; 3 Department of Pathology, Pandit Deendayal Upadhyay Memorial Health Science and Ayush University of Chhattisgarh, Raipur, IND; 4 Department of General Medicine, Mahatma Gandhi Mission (MGM) Medical College, MGM Institute of Health Sciences (MGMIHS), Aurangabad, IND; 5 Department of Pathology, Adesh Medical College and Hospital, Mohri, IND; 6 Department of Naturopathy, Maharishi Aurobindo Subharti College and Hospital of Naturopathy and Yogic Sciences, Swami Vivekanand Subharti University, Meerut, IND; 7 Department of Internal Medicine, Smt. B. K. Shah Medical Institute and Research Centre, Vadodara, IND

**Keywords:** care coordination, general medicine, health system integration, multimorbidity, whole-person care

## Abstract

Multimorbidity has become a major challenge for contemporary healthcare systems, as patients with multiple chronic conditions often require care that extends beyond disease-specific pathways. Fragmented services, polypharmacy, treatment burden, and poor continuity can limit the effectiveness of conventional clinical models. This review aimed to synthesize original evidence on whole-person care, multimorbidity management, and health system integration, with emphasis on the role of general medicine as a clinical bridge. A systematic search of electronic databases was conducted for original English-language studies published from 2016 onward. Study selection followed the Preferred Reporting Items for Systematic Reviews and Meta-Analyses (PRISMA) approach, and 11 studies were included in the final qualitative synthesis. Data were extracted on study design, population, care model, outcomes, key findings, and relevance to integrated generalist care. No meta-analysis was performed because of heterogeneity in study designs, interventions, and outcome measures. The review found that general practice and primary care interventions commonly used extended consultations, relational continuity, individualized care plans, medication review, self-management support, interdisciplinary collaboration, and cross-sector coordination. Benefits were more consistent for patient-centered experience, emotional support, care coordination, medication-related processes, and health behaviors than for generic quality-of-life outcomes. General medicine appears central to translating fragmented multimorbidity care into coordinated, whole-person, and system-aware clinical practice.

## Introduction and background

Multimorbidity, defined as the presence of two or more chronic conditions within the same person, has become a major challenge in modern clinical practice [1]. Population ageing, improved survival from acute diseases, and the rising prevalence of chronic non-communicable diseases have increased the number of patients requiring long-term, coordinated, and personalized care [2]. Individuals with multimorbidity often experience lower functional status, poorer quality of life, psychological distress, polypharmacy, treatment burden, increased healthcare utilization, and a higher risk of adverse outcomes [3]. The complexity of multimorbidity extends beyond the number of diagnoses and includes interactions among diseases, treatments, symptoms, social circumstances, health system access, and patient priorities [4].

Healthcare delivery often remains fragmented by disease, clinical pathway, and episodic service contact [5]. Such fragmentation is poorly aligned with the needs of patients whose care spans primary care, specialist services, community care, rehabilitation, pharmacy, and social support. Disease-specific guidance may be helpful for individual conditions, but it can also contribute to conflicting advice, duplicated investigations, medication-related harm, and excessive treatment burden in patients with multimorbidity [3,6]. General medicine and primary care are therefore positioned as key coordinating disciplines that can integrate biomedical, functional, psychological, and social dimensions of care.

Whole-person care has developed as a response to the limitations of disease-centered medicine. It focuses on the individual rather than the condition alone and includes patient preferences, shared decision-making, emotional support, family involvement, treatment prioritization, self-management support, and continuity [7]. In multimorbidity, this approach requires a shift from disease-centered care to care that is guided by health priorities, functional status, medication burden, and quality of life [8]. Because generalist clinicians often provide longitudinal care and coordinate across complex health systems, they are well placed to operationalize whole-person care for patients with multiple chronic conditions [9].

Health system integration is closely linked to whole-person multimorbidity care. Integration includes interprofessional communication, shared care plans, medication reconciliation, role clarification, timely information sharing, and follow-up after specialist or hospital contact [10]. Care models designed to support this role have included longer consultations, named general practitioners, pharmacist-led medication review, interdisciplinary case conferences, structured self-management support, health coaching, and patient-centered care improvement programs [11]. Together, these approaches reflect a shift from disease-specific and reactive care toward coordinated, proactive, and person-centered systems of care.

Despite increasing interest in whole-person and integrated care, uncertainty remains about which models produce meaningful benefits for patients with multimorbidity [10]. Previous research has reported inconsistent outcomes. Some interventions improve patient experience, care coordination, self-management, medication safety, emotional support, or health behaviors, whereas generic quality-of-life measures often show neutral effects [12]. This variation indicates an evidence gap regarding how general medicine functions as the clinical link between patient complexity and health system fragmentation. There is also uncertainty about the relative contribution of interdisciplinary care, task-shifting, longer consultations, written care plans, and medication review as core components of multimorbidity care.

A focused synthesis is needed to clarify the role of general medicine in whole-person care, the structure of multimorbidity interventions, the outcomes most likely to improve, and the implementation barriers that influence effectiveness. Such a synthesis can help define generalist care as both a clinical and system-level activity that connects patient priorities with coordinated service delivery. Including randomized, mixed-methods, qualitative, feasibility, economic, and quasi-experimental studies allows broader interpretation of effectiveness, patient experience, feasibility, and mechanisms of integration.

Objectives of the review

The objective of this review was to synthesize original evidence on whole-person care, multimorbidity management, and health system integration within general medicine and primary care settings. The review aimed to identify key care models, patient-centered and system-level outcomes, coordination mechanisms, and implementation patterns relevant to general medicine as a clinical bridge for patients with multimorbidity.

## Review

Methodology

Search Strategy

A systematic search was conducted using electronic databases including PubMed/MEDLINE, Scopus, Embase, Web of Science, and Google Scholar to identify original studies relevant to general medicine, whole-person care, multimorbidity, patient-centered care, primary care coordination, and health system integration. The search focused on studies published from 2016 onward to ensure that the included evidence reflected contemporary models of multimorbidity care and integrated clinical practice. The final search was updated during manuscript revision. Search terms were combined around concepts such as "multimorbidity", "whole-person care", "patient-centred care", "general practice", "primary care", "care coordination", "integrated care", "interdisciplinary care", and "health system integration". A representative search string was as follows: (“multimorbidity” OR “multiple chronic conditions” OR “complex chronic disease”) AND (“whole-person care” OR “patient-centred care” OR “person-centred care”) AND (“general practice” OR “primary care” OR “family medicine” OR “general medicine”) AND (“care coordination” OR “integrated care” OR “interdisciplinary care” OR “health-system integration”). The study selection process followed the Preferred Reporting Items for Systematic Reviews and Meta-Analyses (PRISMA) approach [13]. Titles and abstracts were screened for eligibility, followed by full-text assessment of potentially relevant articles. Eligibility decisions were based on predefined inclusion and exclusion criteria, and uncertainties were resolved through reviewer discussion. A total of 252 records were identified through database searching, and after duplicate removal, title-and-abstract screening, and full-text assessment, 11 studies were included in the final qualitative synthesis, as detailed in the PRISMA flow diagram. To ensure the currency and relevance of the evidence base, major recent systematic reviews on multimorbidity interventions, integrated care models, and patient-centered multimorbidity care were reviewed for contextual relevance. Because the present review focused on original studies, systematic reviews were not included in the final qualitative synthesis but were used to strengthen the Background and Discussion sections where appropriate.

Eligibility Criteria

Inclusion criteria: Studies were eligible if they were original research articles published in English between 2016 and the present that dealt with adults with multimorbidity or complex chronic disease needs within general medicine, primary care, or family medicine or integrated care settings. Studies included in the review addressed whole-person care, patient-centered care, care coordination, interdisciplinary care, medication review, support for self-management, extended consultations, or health system integration. Randomized trials, pragmatic trials, mixed-methods studies, qualitative studies, feasibility studies, quasi-experimental studies, and economic evaluations were eligible if they reported relevant clinical, patient-reported, implementation, coordination, and/or system-level outcomes.

Exclusion criteria: The studies were not included in the final evidence synthesis if they were review articles, systematic reviews, scoping reviews, narrative reviews, editorials, commentaries, protocols, conference abstracts, opinion papers, published in a language other than English, published before 2016, or lacking sufficient information on outcomes. Studies that did not include a component on multimorbidity or integrated care were excluded. Articles that consisted of review articles, systematic reviews, scoping reviews, and narrative reviews were included in the Background, Conceptual framing, and, for comparative purposes, in the Introduction and Discussion, as appropriate, but are not included in the Results synthesis.

Data Extraction and Analysis

The data were obtained in a systematic way according to the characteristics and outcomes of each included study. Data extraction was performed using a predefined extraction framework to ensure consistency across studies. Information extracted consisted of author and year, study design, care setting, population characteristics, multimorbidity definition, sample number, intervention or care model, comparator if provided, outcomes measured, key findings, and relevance to whole-person care and health system integration. Extracted information was checked for completeness and consistency, and any uncertainty regarding eligibility, extracted data, or interpretation was resolved through discussion among the review authors.

The findings were compiled into a key characteristics and outcomes table in the Results section. The synthesis was qualitative, and no meta-analysis was conducted because the included studies differed substantially in study design, population, intervention type, comparator, outcome measures, follow-up period, and reporting structure. Themes were synthesized by grouping findings according to recurring care components and outcome domains across studies. Numerical findings, including effect estimates, confidence intervals, p-values, and economic outcomes, were summarized descriptively where available and were not statistically pooled. Findings were synthesized in thematic sections of whole-person care, patient-centered outcomes, care coordination, interdisciplinary care, task-shifting, implementation barriers, medication safety, and health system integration.

Quality Assessment

Studies included were evaluated based on the study design and clarity of study aims, methodology, description of population, description of intervention, reporting of outcomes, follow-up, and relevance to the review question. Randomized and pragmatic trials were evaluated for randomization, methods of allocation, delivery of the intervention, completeness of outcomes, and group comparability. Studies that used qualitative or mixed-methods data were evaluated for the suitability of sampling, clarity of data collection, transparency of analysis, and theme relevance to care of multimorbidity data. The methods, outcome reporting, and suitability of economic and feasibility studies to support system-level interpretation were evaluated. Overall, the evidence presented was considered appropriate for qualitative synthesis, except for a few studies, which lacked pragmatic implementation, small sample size, short follow-up period, or context-specific care models.

Risk of Bias Assessment

The level of risk of bias was determined by conventional design-specific tools: RoB 2 (Risk of Bias 2) [14] was used for randomized studies, and ROBINS-I (Risk Of Bias In Non-randomised Studies - of Interventions) [15] was used for non-randomized studies. The majority of studies were rated as moderate risk of bias due to the difficulty of being blinded, the use of real-world clinical trials, a relatively short follow-up time, and/or differences in implementation between practices or care teams. Randomized trial studies typically offered more robust evidence for effectiveness, and although qualitative and mixed-methods studies did not provide as strong evidence for effectiveness, they did offer valuable insights related to patient experience, coordination mechanisms, and implementation challenges. Studies with a small feasibility sample size and studies that lacked a comparator were interpreted with caution and were used primarily to support acceptability, feasibility, and system integration findings, rather than definitive effectiveness findings.

Results

Search Results and Study Selection

**Figure 1:**
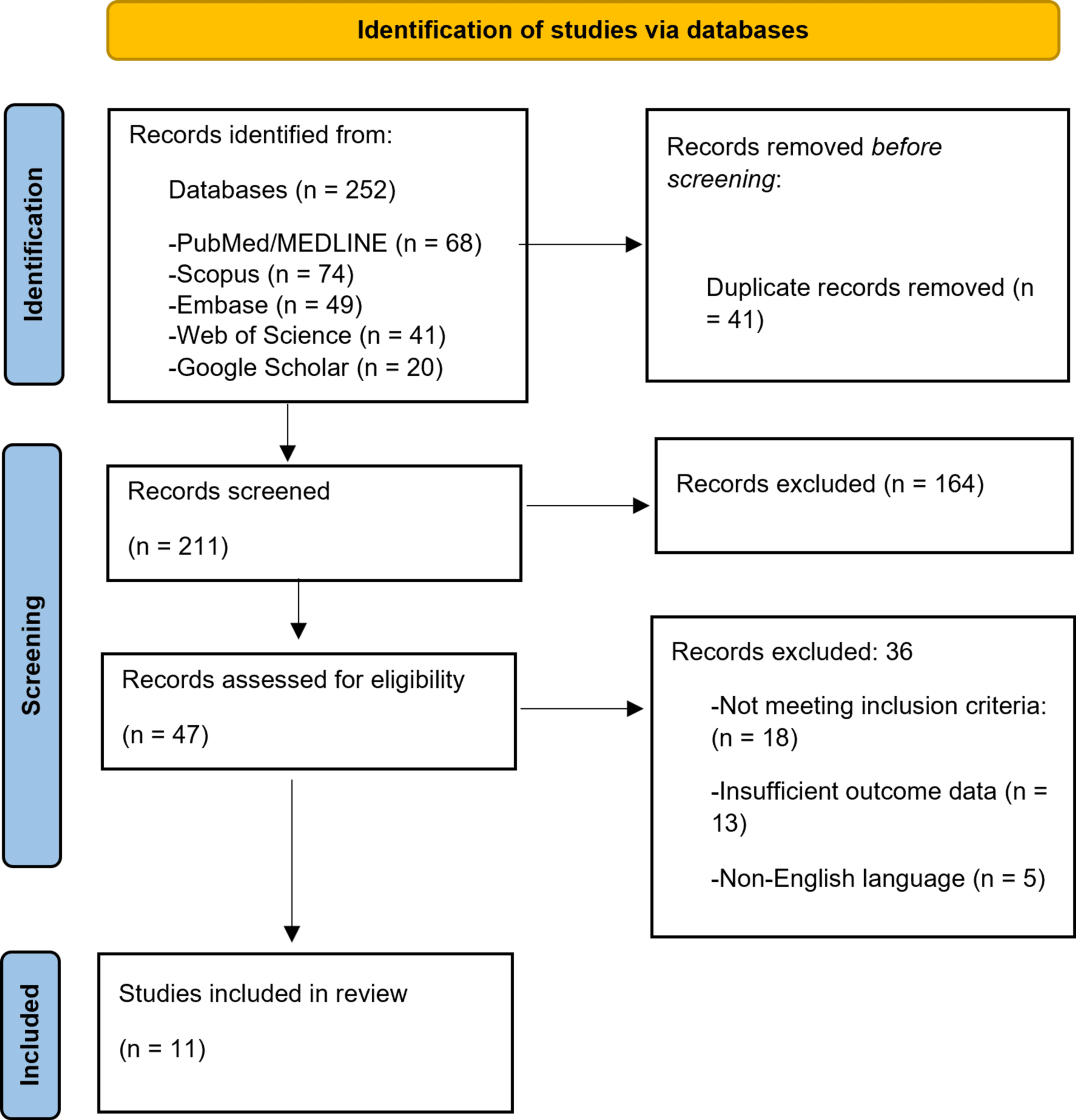
PRISMA flowchart PRISMA: Preferred Reporting Items for Systematic Reviews and Meta-Analyses

The database search identified 252 records across the included databases: PubMed/MEDLINE (n=68), Scopus (n=74), Embase (n=49), Web of Science (n=41), and Google Scholar (n=20). After the removal of 41 duplicate records, 211 records were screened by title and abstract. Of these, 164 records were excluded because they did not meet the review eligibility criteria. A total of 47 full-text articles were assessed for eligibility, and 36 articles were excluded for the following reasons: not meeting the inclusion criteria (n=18), insufficient outcome data (n=13), and non-English language (n=5). Eleven original studies were included in the final qualitative synthesis. The selected studies provided evidence to explore whole-person care, multimorbidity management, and the involvement of general medicine in health system integration. This revised reporting provides a clearer account of the study selection pathway in accordance with the PRISMA approach and corresponds to the flow diagram presented in Figure 1.

Study Characteristics

The studies included were published from 2016 to 2021 and are largely based in general practice, family medicine, primary care, and cross-sector care settings. Pragmatic and cluster randomized trials, mixed-methods studies, qualitative investigations, a feasibility study, an economic evaluation, and a quasi-experimental analysis were included in the evidence base. Study populations included adults with multimorbidity, typically defined as having two or more or three or more chronic conditions. These care models across studies included extended consultations, relational continuity, individualized care plans, medication review, self-management support, interdisciplinary working, and healthcare sector coordination. Details of characteristics and outcome findings of the included studies are summarized in Table 1.

**Table 1:** Key characteristics and outcomes of the included studies EQ-5D-5L: EuroQol Five-Dimension Five-Level Questionnaire; W-BQ12: 12-Item Well-Being Questionnaire; HADS: Hospital Anxiety and Depression Scale; QALYs: quality-adjusted life years; AUC: area under the curve; GP: general practitioner; NHS/PSS: National Health Service/Personal Social Services; ICER: incremental cost-effectiveness ratio; heiQ: Health Education Impact Questionnaire; SE-CD: Self-Efficacy for Managing Chronic Disease Scale; PCC: patient-centered care; MPA: medical practice assistant; COPD: chronic obstructive pulmonary disease; PACIC: Patient Assessment of Chronic Illness Care; EQ-5D-3L: EuroQol Five-Dimension Three-Level Questionnaire; VR-12: Veterans RAND 12-Item Health Survey; EQ-5D: EuroQol Five-Dimension Questionnaire

Study	Design	Sample/population	Intervention or care model	Comparator	Outcomes assessed	Main findings	Result section interpretation
Mercer et al. [16]	Phase 2 exploratory cluster randomized controlled trial with cost-utility analysis	8 general practices; 152 patients aged 30-65 years with ≥2 long-term conditions; mean age about 52 years; mean of about five chronic conditions; most lived in highly deprived areas	CARE Plus: whole-system primary care intervention involving 30-45 minute consultations, relational continuity, practitioner training/support, holistic assessment, patient priorities, care planning, self-management materials, and links to local/community resources	Usual care	EQ-5D-5L quality of life; W-BQ12 well-being; HADS anxiety/depression; self-efficacy; self-esteem; health service use; QALYs; cost-effectiveness	CARE Plus improved EQ-5D-5L at six months but not significantly at 12 months; EQ-5D-5L AUC over 12 months favored CARE Plus; negative well-being improved significantly at 12 months; cost per QALY gained was £12,224, with a high probability of cost-effectiveness at common UK thresholds	Supports the role of enhanced general practice as a whole-person care bridge in deprived multimorbid populations. The strongest outcome signal was preservation/improvement of quality of life and negative well-being rather than broad gains across all psychosocial outcomes
Salisbury et al. [17]	Pragmatic cluster randomized trial	33 practices; 1546 adults with ≥3 chronic conditions: 797 intervention and 749 usual care	3D model: six-monthly comprehensive review addressing dimensions of health, depression, and drugs; nurse review, pharmacist medication review, named GP review, individualized health plan, continuity promotion, electronic templates, staff training, feedback, and financial incentives	Usual care	Primary: EQ-5D-5L quality of life at 15 months. Secondary: illness burden, treatment burden, patient-centered care, continuity, consultation use, disease-specific care indicators, high-risk prescribing	No difference in quality of life at 15 months: adjusted EQ-5D-5L difference 0.00, 95% CI: -0.02 to 0.02, and p=0.93. Outcomes included illness burden, treatment burden, patient-centered care, continuity, care processes, and prescribing safety	A large, well-designed test of a generalist multimorbidity review did not improve generic quality of life. It remains important because it operationalized whole-person care within routine general practice and demonstrated the difficulty of translating patient-centered redesign into measurable quality-of-life gain
Thorn et al. [18]	Economic evaluation alongside a pragmatic cluster randomized controlled trial	Same trial population: 797 intervention and 749 usual care adults with ≥3 chronic conditions	Economic evaluation of the 3D intervention: six-monthly GP reviews, nurse appointments, pharmacist medication review, named GP, individualized care plan	Usual care	QALYs; NHS/PSS costs; ICER; cost consequences from NHS/PSS, patient/carer, and productivity perspectives	QALY gain was very small: adjusted mean difference 0.007 and 95% CI: -0.009 to 0.023. Costs were also slightly higher: adjusted mean difference £126 and 95% CI: -£739 to £991. ICER was £18,499/QALY; the probability of cost-effectiveness was 50.8% at £20,000/QALY and 55.8% at £30,000/QALY. Authors judged the cost-effectiveness as equivocal	Use as a health system value evidence. The 3D model was not clearly inefficient, but economic uncertainty was substantial. It shows that integrated multimorbidity care may require better targeting, deprescribing, and replacement of disease-specific reviews rather than the addition of new review layers
Fortin et al. [19]	Pragmatic mixed-methods randomized controlled trial	284 patients aged 18-80 years with ≥3 chronic conditions from seven family medicine groups: 144 intervention and 140 control; the mean number of chronic diseases was 5.0 in both groups	Four-month interdisciplinary intervention delivered by nurses, nutritionists, kinesiologists, and other primary care professionals; motivational approach; self-management support; individualized care pathways; community of practice	Delayed intervention/usual care during the waiting period	Primary: self-management using the heiQ and self-efficacy. Secondary: health status, quality of life, psychological distress, health behaviors. Qualitative interviews with patients, family members, and professionals	Primary outcomes were neutral. One heiQ domain, self-monitoring and insight, improved modestly; SE-CD showed no intervention effect. Physical activity and healthy eating improved significantly; no improvement in health status or quality of life; no adverse events	Demonstrates that interdisciplinary whole-person primary care may improve health behaviors even when generic quality-of-life and self-efficacy scores do not change. Qualitative benefit exceeded quantitative signal, supporting mixed-methods interpretation in multimorbidity research
Kuipers et al. [20]	Constructivist qualitative study	Nine healthcare professionals: four GPs and five nurse practitioners from seven GP practices participating in a PCC improvement program	No clinical intervention tested; explored barriers to delivering patient-centered care across eight PCC dimensions	Not applicable	Perceived barriers across patient preferences, information/education, access, physical comfort, emotional support, family/friends, coordination, continuity/transition	Barriers were found in all eight PCC dimensions. Main barriers included difficulty reaching mutual understanding with patients, lack of training in new PCC skills, data protection constraints affecting documentation/information sharing, time pressure, and conflicting financial incentives	Provides implementation evidence. Whole-person care is not only a clinical communication issue; it depends on organizational capacity, information governance, financing, time, and skill development
Kuipers et al. [21]	Mixed-methods evaluation	Nine interviewed GPs/nurse practitioners; longitudinal survey of 138 patients with multimorbidity who completed baseline and one-year follow-up	PCC improvement program using workshops and an intervention toolbox: listening, motivational interviewing, shared decision-making, teach-back, illiteracy recognition, "three good questions", topic lists, consultation videotaping, and PCC-on-the-job evaluation	Pre-post comparison; no separate control group	Patient-centered primary care instrument; PCC dimensions; healthcare professional experiences	Patient-perceived PCC improved significantly over one year: t=2.66 and p=0.005. Significant improvements were seen in physical comfort, emotional support, continuity/transition, and family/friends; coordination improved only marginally; patient preferences, access, and information/education did not significantly improve. The intervention toolbox targeted all eight PCC dimensions	Supports practice-level PCC improvement as a feasible route for whole-person care. Gains were strongest in relational/supportive dimensions rather than structural access or information domains
Shah et al. [22]	Quasi-experimental difference-in-differences analysis	National GP patient survey data from 3.5 million respondents, 2013-2017; main analysis focused on multimorbid patients	Enhanced primary care model adding non-medical health coaches to primary care teams to support prevention, self-management, patient activation, and single-point contact	Comparable GP practices in the rest of England	Health status, EQ-5D-5L domains, physical functioning, psychological well-being, resilience, smoking, person-centeredness, continuity, primary care use	Among multimorbid patients, psychological well-being declined slightly: -0.0174 and 95% CI: -0.0283 to -0.0065. Person-centeredness also declined short term: -0.0356 and 95% CI: -0.0530 to -0.0183. No consistent effects appeared for other multimorbid outcomes. At the population level, primary care utilization declined by -0.0331 and 95% CI: -0.0448 to -0.0214	Task-shifting to non-medical health coaches may reduce utilization, but does not automatically improve patient experience or health outcomes. For whole-person care, workforce redesign must preserve relational trust and continuity
Stumm et al. [23]	Qualitative interview study	32 interviews: 16 GPs and 16 medical practice assistants from 16 practices	No clinical intervention; explored GP/MPA perspectives on coordination and possible navigator/support roles	Not applicable	Coordination tasks, barriers, administrative burden, cross-sector communication, delegation, navigator models	GPs viewed themselves as central coordinators and described coordination as linking medical, social, legal, family, and community resources. Barriers included administrative burden, regulatory constraints, poor communication with external providers, delayed hospital discharge information, lack of time, and inadequate remuneration. GPs preferred support from an additional qualified person within the practice team rather than an external navigator	Strongly supports the review concept of general medicine as the clinical bridge. General practice acts as the system integrator, but coordination is undermined by fragmented communication, bureaucracy, and under-resourced care coordination
Birke et al. [24]	Mixed-methods feasibility study	48 patients; mean age: 72.2 years; ≥2 of diabetes, COPD, and cardiovascular disease; all had hospital contact in the previous year	Complex intervention for multimorbidity: extended GP consultations up to 60 minutes, nurse care manager, individualized care plan based on patient goals, cross-sector coordination, medication review, rehabilitation referral, potential shifting of outpatient visits to general practice, electronic sharing of care plan	No control group	Feasibility, acceptability, PACIC, EQ-5D-3L, focus groups, observations	Patients and professionals found the intervention acceptable. 54% were referred to municipal rehabilitation and 29% to medication review, 13% discontinued hospital outpatient control visits, and 79% completed the second consultation. Patient themes: prior lack of cross-sector coordination, better coordination through extended consultations, and desire for involvement in care planning	Supports the feasibility of general practice-led integrated care. Extended consultations and care manager coordination helped reposition general practice as the hub between hospital, municipality, rehabilitation, medication review, and patient goals
Pohontsch et al. [25]	Qualitative focus group study with quality indicator development	Eight patient focus groups and three relative focus groups; 47 patients aged 65-84 years with ≥3 chronic conditions; nine relatives	No clinical intervention; patient/relative-informed development of quality indicators for multimorbidity care	Not applicable	Patient-relevant quality aspects; quality indicators; patient education, medication plans, biopsychosocial needs, shared decision-making, family involvement, medication review	Four new patient-derived quality indicators were created; two were accepted by the expert panel: patient education/self-management and regular medication plan updates. Patient accounts supported biopsychosocial assessment, family/caregiver involvement, shared decision-making, treatment goal agreement, medication information, medication review, adverse drug reaction documentation, and written treatment plans	Provides patient-defined outcome domains for whole-person care. The Results section can use this study to justify why outcomes should include self-management education, medication safety, written care plans, caregiver involvement, and biopsychosocial assessment rather than disease metrics alone
Stewart et al. [26]	Mixed-methods pragmatic randomized trial and qualitative study	163 randomized patients aged 18-80 years with ≥3 chronic conditions: 86 intervention and 77 control; qualitative subsample of 14 patients	Multi-provider case conference planned by nurse and patient; included family physician, internist, psychiatrist, social worker, physiotherapist, occupational therapist, pharmacist, dietitian, home care case manager as needed; patient goals and agreed care plan; nurse follow-up for four months	Usual family physician care plus a list of community resources	Primary: heiQ and self-efficacy. Secondary: VR-12 physical/mental health, EQ-5D, psychological distress, health behaviors. Qualitative patient experience	No significant primary outcome differences overall. Mental health improved only in patients with an annual income of ≥C$50,000: β=11.003 and p=0.006. More providers and ≥3 hours of nurse follow-up were associated with poorer outcomes. Qualitative themes: valuing the team, feeling supported, receiving a follow-up plan, receiving helpful treatment additions, and experiencing positive outcomes	Shows both promise and risk in high-complexity integrated care. Multi-provider conferences can validate patients and generate useful plans, but excessive team complexity may increase burden unless care plans are actionable, prioritized, and financially accessible

Risk of Bias Assessment

The risk of bias was evaluated using conventional tools for design-appropriate studies, with RoB 2 for randomized trials and ROBINS-I for non-randomized trials. The majority of studies included were scored at the moderate level of risk of bias, primarily due to pragmatic intervention implementation, unblinding, brief intervention duration, sample size, or local implementation of the intervention. Randomized designs generally provided high internal validity, and non-randomized and qualitative studies were predominantly utilized to support the interpretation of care coordination, patient experience, and barriers to implementation. Detailed judgments are shown in Table 2.

**Table 2:** Risk of bias assessment of the included studies RoB 2: Risk of Bias 2; ROBINS-I: Risk Of Bias In Non-randomised Studies - of Interventions

Study	Tool used	Main concern	Risk of bias
Mercer et al. [16]	RoB 2	Small number of clusters; exploratory design; no participant blinding	Moderate
Salisbury et al. [17]	RoB 2	Pragmatic delivery; no participant or practice blinding	Low
Thorn et al. [18]	ROBINS-I	Economic analysis dependent on trial data; uncertainty in cost estimates	Moderate
Fortin et al. [19]	RoB 2	Short follow-up; blinding not feasible	Moderate
Kuipers et al. [20]	ROBINS-I	Small provider sample; context-specific findings	Moderate
Kuipers et al. [21]	ROBINS-I	No control group; attrition risk	High
Shah et al. [22]	ROBINS-I	Non-randomized design; possible residual confounding	Moderate
Stumm et al. [23]	ROBINS-I	Provider-only perspective; context-specific setting	Moderate
Birke et al. [24]	ROBINS-I	Small feasibility sample; no comparator	High
Pohontsch et al. [25]	ROBINS-I	Focus group evidence; indirect outcome assessment	Moderate
Stewart et al. [26]	RoB 2	Modest sample size; partial intervention non-receipt	Moderate

Whole-Person Care and Patient-Centered Outcomes

**Figure 2:**
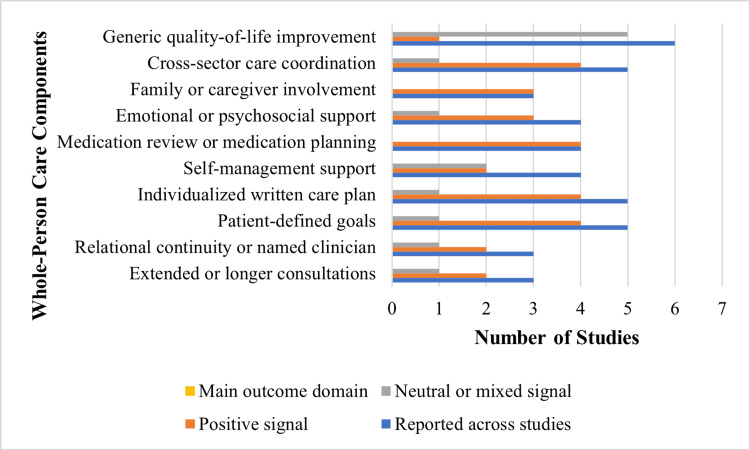
Distribution of whole-person care components and outcome direction

Whole-person care was evidenced by the redesign of consultations, continuity, patient-defined goals, individual care planning, self-management support, emotional care, family involvement, and biopsychosocial needs. Patient-centered and relational outcomes were more responsive than generic quality-of-life measures throughout the evidence base. Most frequently reported improvements were in perceived support, emotional care, self-monitoring, health behaviors, care planning, and care coordination experience. The relation, functional, and integration-focused benefits of whole-person care in multimorbidity were less consistently captured with generic health-related quality of life, indicating a need for tools that better reflect the relational aspects of care in multimorbidity. The frequencies and directions of the whole-person care components reported in the included studies are summarized in Figure 2.

Multimorbidity Care Coordination and Health System Integration

**Figure 3:**
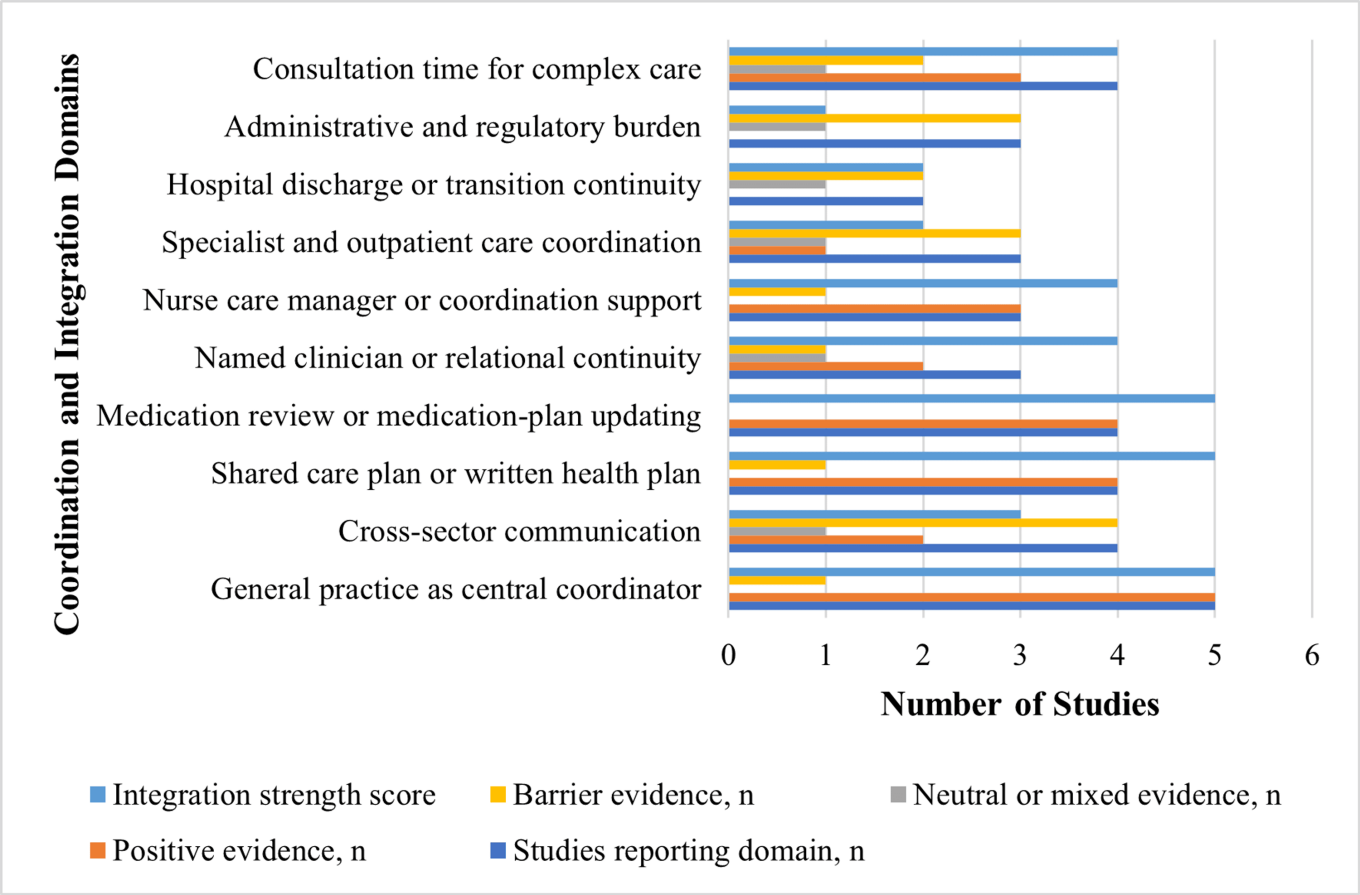
Care coordination and health system integration domains across the included studies

Coordination of care was the most common system-related theme identified among the included studies. General medicine was highlighted multiple times as the fulcrum point of connecting disease-specific care with the need for patient-centered, integrated, and continuous care. Coordination was enabled through longer consultations, a specific clinician, a nurse care coordinator, medication assessment, care planning, and cross-sector communication. The main obstacles included a lack of information flow, poor communication between specialists and primary care, administrative constraints, short consultations, and poor follow-up after inpatient or outpatient care. Figure 3 illustrates the distribution of coordination domains, supporting evidence, barrier evidence, and integration strength across the included studies.

Discussion

This discussion interprets the main findings of the review and focuses on their implications for general medicine, whole-person care, multimorbidity management, and health system integration. The current systematic review investigated the role played by general medicine in acting as a clinical interface for whole-person care, multimorbidity management, and health system integration. The findings from the literature review suggest that the management of patients with multimorbid conditions occurs primarily within general practices and primary care centers. Throughout the literature, the commonest observation made was not an increase in general health-related quality-of-life measures, but rather benefits observed in aspects such as coordination of care, patient communication, emotional support, medication management, self-management support, and continuity of care. This implies that the impact of whole-person care may be felt more significantly through relational and organizational processes than by the use of standardized measures of health status.

The findings of this review are consistent with recent systematic review evidence on multimorbidity and integrated care. Recent reviews have reported that multimorbidity interventions in primary care and community settings show variable effects on broad patient-reported outcomes, health-related quality of life, mental health, and service-use outcomes. This pattern supports the interpretation that the effectiveness of whole-person and integrated care models depends on intervention structure, population complexity, implementation context, and outcome selection. Reviews of integrated care and co-designed multimorbidity interventions have also emphasized heterogeneity in care components, delivery settings, professional roles, and evaluation measures. These findings strengthen the present synthesis by indicating that whole-person and integrated care approaches are clinically relevant, but their benefits may be better captured through patient experience, coordination, treatment burden, medication safety, and continuity rather than generic quality-of-life measures alone [27-29].

One major insight was that a whole-person approach to healthcare needed a paradigm shift from a disease-oriented perspective to a patient-focused one. The interventions that included CARE Plus, the 3D framework, complex consultations for multimorbidity patients, and multi-provider case conferences were intended to address the limitations of disease-oriented management by considering factors like patients' objectives, drug safety, the burden on the patient due to various treatments, and social aspects. This is supported by existing literature, which suggests that multimorbidity cannot be effectively managed using guidelines that focus on a single disease because such an approach may exacerbate issues like polypharmacy and increase consultation load [30].

The findings further reveal that general medicine serves as the coordinating basis for the care of people with multimorbidities. Research examining care coordination highlighted the fact that people suffering from many chronic diseases tend to move between general practice, specialists, hospital services, community-based services, rehabilitation services, and even social services. At each stage of such a journey, the general practitioner or the team serving as the primary care provider tends to become the only constant source of continuity in terms of clinical care. Such a trend is consistent with health system literature that regards primary care as the building block for integrated care systems [28], especially when people need ongoing care and support.

Medication review became a key component of multimorbidity care integration. Pharmacist review, medication plan modification, and the evaluation of treatment burden were included in some of the relevant studies. Indeed, the complexity of the medication regimen, drug interaction, adverse effects, duplication, and misunderstanding of the treatment process often arise in clinical practice due to multimorbidity. Polypharmacy and medication burden have also been considered significant issues in multimorbidity by previous literature [31]. In the current systematic review, however, medication planning was both a biomedical concern and an issue concerning communication, education, continuity, and self-management. Modification of the medication plan and the explanation of its intended purpose were particularly relevant.

It is significant to note the mixed results pattern obtained from the studies. Generic quality of life tended to have no positive effect on large pragmatic trials, but there were significant improvements in patients' experiences, sense of support, coordination of care, and engagement according to qualitative and mixed-methods research. This trend was in line with past evidence on how tools for assessing generic quality of life likeEuroQol Five-Dimension Questionnaire (EQ-5D) may fail to adequately gauge the impact of patient-centered and integrated care models, especially interventions focused on communications, confidence, care burden, and care navigation [32]. Overall quality of care for the whole individual may be better gauged by patient experience, care burden, self-care confidence, and medication safety.

Team-based care was yet another recurring topic, but the available literature indicates that it needs to be structured properly to be effective. Indeed, nurses, pharmacists, health coaches, social workers, rehabilitators, and care managers may help with improving the quality of care planning and coordination. Nevertheless, involving new players does not necessarily result in better results. There were a few studies demonstrating no changes in the quality of quantitative results, and there was even one intervention by means of health coaching that resulted in little positive effect and some negative trends in patient-centeredness and psychological well-being of patients. It is consistent with the ongoing conversation about the need for role definition, relationship continuity, and patients' acceptance for the model of care to work [33].

Equity was another theme present in the results of the research. First, CARE Plus research showed that increased primary care could be beneficial for socioeconomically deprived populations, whereas one study pointed out the differences in the effect of treatment for those with different incomes. This evidence confirms what is already known about multimorbidity occurring earlier in life, causing greater distress, and being more complicated in socially deprived groups [10]. Thus, whole-person care has to account not only for the complexities in treatment but also for access issues, affordability, patients' needs, and other aspects. On balance, the review reveals general medicine as a way of joining patient complexity and healthcare system fragmentation. Multimorbidity whole-person care should be viewed through the lens of the model, incorporating biomedical treatment, psychosocial perspective, patient safety, patients' needs and goals, and collaboration among professionals. The value of general medicine should thus be sought in its ability to integrate these components into the clinical process.

Limitations and Future Recommendations

This review has several limitations. These studies were heterogeneous in terms of study design, patient population, structure of the intervention, outcome variables, and follow-up period, thus preventing meta-analysis and calling for the qualitative synthesis of the results. A number of the studies employed a pragmatic or mixed-methods approach to research, whereby blinding was not easy, and the mode of delivery of the intervention differed from one setting to another. A few other studies featured small sample sizes, short follow-up periods, or specialized care approaches, which might limit their external validity. Although recent systematic reviews were considered during revision to confirm the currency and contextual relevance of the literature, the qualitative synthesis was restricted to original studies in accordance with the eligibility criteria.

Future studies need to implement interventions that have been well developed and validated and to utilize appropriate outcome measures that include patient-centered experiences, treatment burden, care coordination, medication safety, and quality of life. There is a need for larger multicenter randomized controlled trials with longer follow-ups to establish the efficacy of general medicine-based integrated care. Future studies should investigate the effect of socioeconomic factors, health literacy, caregivers' participation, and availability of coordinated care on the outcomes of integrated care.

## Conclusions

From this systematic review, it can be said that general medicine plays a vital role in bridging disease-specific therapy, patient needs, pharmacological safety, psychosocial factors, and health system coordination in providing whole-person care to individuals with multimorbidity. In general practice and primary care interventions from the selected articles, there were extended consultations, continuity of relationship, tailored care plan, medication management, self-care, teamwork, and communication between different sectors. Positive changes in patient-centeredness, support perception, coordination of care, emotional support, medication process, and health behaviors were observed in contrast to quality of life, which was usually unchanged. Thus, it is suggested that the benefits of whole-person multimorbidity care might be beyond the scope of traditional biomedical or general health status measures. From the research, it is evident that integration requires sufficient consultation time, clarification of roles, sharing of information, continuity of care, and follow-up. It can be concluded that general medicine offers a framework for whole-person multimorbidity care implementation within healthcare systems.
